# Embracing context: Lessons from designing a dialogue-based intervention to address vaccine hesitancy

**DOI:** 10.3389/fpubh.2023.1069199

**Published:** 2023-02-20

**Authors:** ToTran Nguyen, Lise Boey, Carla Van Riet, Stef Dielen, Hélène Dodion, Tamara Giles-Vernick, Nico Vandaele, Heidi J. Larson, Koen Peeters Grietens, Charlotte Gryseels, Leonardo W. Heyerdahl

**Affiliations:** ^1^Socio-Ecological Health Research Unit, Department of Public Health, Institute of Tropical Medicine, Antwerp, Belgium; ^2^Department of Work and Organisation Studies, KU Leuven, Leuven, Belgium; ^3^Access-To-Medicines Research Centre, KU Leuven, Leuven, Belgium; ^4^Anthropology and Ecology of Disease Emergence Unit, Department of Global Health, Institut Pasteur, Paris, France; ^5^Vaccine Confidence Project, London School of Hygiene and Tropical Medicine, London, United Kingdom; ^6^School of Tropical Medicine and Global Health, Nagasaki University, Nagasaki, Japan

**Keywords:** dialogue, COVID-19, vaccine hesitancy, dialogue-based intervention, digital intervention, participatory research, public health intervention

## Abstract

Dialogue with people who are vaccine hesitant has been recommended as a method to increase vaccination uptake. The process of cultivating dialogue is shaped by the context in which it occurs, yet the development of interventions addressing vaccine hesitancy with dialogue often overlooks the role of context and favors relatively fixed solutions. This reflexive paper shares three key lessons related to context for dialogue-based interventions. These lessons emerged during a participatory research project to develop a pilot intervention to create open dialogue among healthcare workers in Belgium about COVID-19 vaccination concerns. Through a mixed methods study consisting of in-depth interviews, focus group discussions, and surveys, we engaged healthcare workers in the design, testing, and evaluation of a digital platform featuring text-based and video-based (face-to-face) interactions. The lessons are: (1) what dialogue means, entails, and requires can vary for a population and context, (2) inherent tension exists between helping participants voice (and overcome) their concerns and exposing them to others' ideas that may exacerbate those concerns, and (3) interactional exchanges (e.g., with peers or experts) that matter to participants may shape the dialogue in terms of its content and form. We suggest that having a discovery-orientation—meaning to work not only inductively and iteratively but also reflexively—is a necessary part of the development of dialogue-based interventions. Our case also sheds light on the influences between: dialogue topic/content, socio-political landscape, population, intervention aim, dialogue form, ethics, researcher position, and types of interactional exchanges.

## 1. Introduction

The COVID-19 pandemic raised awareness of the necessity of dialogue for addressing vaccine concerns (1–7). Considered an effective approach for overcoming vaccine hesitancy (8), dialogue is an important way to learn how context, such as the evolving crisis in relation to local socio-cultural and practical complexities, shapes COVID-19 vaccine hesitancy (9, 10). Accordingly, some interventions [e.g., (1, 3, 11–15)] have focused on using dialogue to understand and respond to people's vaccination concerns, with the aim of increasing uptake.

Attending to context can also reveal how to effectively cultivate and ultimately scale dialogue within a population. Intervention efforts to create dialogue spaces to address COVID-19 vaccine hesitancy, however, are limited in the ways they consider and incorporate context (1, 3, 11–15). Relatively fixed approaches and solutions are implemented, reducing the opportunity to understand what could better generate dialogue in evolving contexts. For example, in Knight et al.'s (1) “linear” approach to developing “therapeutic dialogues” that address “the most common vaccine concerns” (p. 99), the COVID-19 pandemic and vaccination context appears as a factor shaping the content of participants' concerns but not as a factor shaping the intervention process itself (e.g., the type/form of dialogue).

From our experience designing and implementing a pilot dialogue-based intervention for addressing COVID-19 vaccine hesitancy, we share three critical lessons that emerged from embracing context. This was a participatory research project to cultivate open dialogue among Belgian healthcare workers (HCWs), which was conducted from November 2021 to March 2022. At the time the project started, there were 1,380,343 confirmed cases of COVID-19 and 26,224 deaths in Belgium (16), significant for a population of 11.5 million (17). The national COVID-19 vaccination campaign kicked off on December 28, 2020 with a prioritizing scheme that began with the residents of nursing homes and HCWs (18). During our project, polarization (in Belgium and globally) was evident between supporters and opponents of the COVID-19 measures, including vaccination (19, 20). The Belgian government's proclamation of mandatory vaccination for HCWs occurred just before the start of our study (21), and this was followed by the last Delta-wave, then the first Omicron-wave (16), which further burdened HCWs. Later, the deadline for mandatory vaccination was postponed until summer 2022 (22) and then eliminated (23), soon after our study concluded. Through a reflexive account, we shed light on the complexities of developing our intervention in this polarizing, evolving context.

Our international, interdisciplinary research team had studied vaccine hesitancy in Flanders, Belgium for a year prior to this project, during which time we had documented polarization online on social media. We had observed “unspoken vaccine hesitancy,” the phenomenon where “health professionals [both vaccinated and not vaccinated] often do not voice their vaccine-related concerns, particularly to colleagues, due to the institutional and societal pressures to vaccinate” (p. 1) (24). That led us to launch this study.

## 2. Our project and intervention design

Our project aimed to mitigate “unspoken vaccine hesitancy” among HCWs through learning how to create open dialogue in a group with varying vaccine sentiments. We saw the intervention as a way to contribute to building vaccine confidence, even though it was not about resolving specific vaccination concerns. To maximize the potential for HCW engagement—given the polarization concerning mandatory vaccination and the pressures of this period for them—we chose to use a digital (online) platform to allow them to engage anonymously and asynchronously.

We conducted in-depth interviews (1-h) and focus group discussions (2-h) with 74 healthcare workers from Flanders and Wallonia (recruited through purposive sampling) to understand three key topics: their COVID-19 vaccination perceptions and concerns, what they experienced as the atmosphere of vaccine discussions, and what they saw as essential features of a safe space for open dialogue among HCWs. The methodology for that, the characteristics of those participants, and the findings about the first two topics, can be found in our previously published paper (25), while this paper focuses on the third topic. Most of these exchanges took place before the launch of the platform, and thus they were not only a source of input into the design of the platform but also a source of potential platform users. Research team members held weekly meetings to discuss findings. Detailed meeting notes from these exchanges were also used as source material for our analysis. Transcripts of focus group discussions and in-depth interviews and meeting notes were imported into Nvivo (QSR international) and coded thematically.

A text-based platform offers users the opportunity to engage with each other asynchronously. At the time of the intervention, offering only a non-text or live platform would have made it extremely difficult to recruit and coordinate with HCWs, given the strain of the pandemic and also the variation in healthcare professions (e.g., their work demands or hours).

For rapid benchmarking, five prominent text-based social media and instant messaging platforms were selected, based on robustness of features for online dialogue and/or popularity: Facebook groups, Reddit, Discord, Slack, and WhatsApp. Criteria for evaluation included the following, which easily eliminated most of the platforms: ease of access and use for users, anonymity from users, flow of conversation (synchronous vs. asynchronous), possibility of sharing links, ease of doing polls and surveys, future value for user, possibility to validate an information sheet (regarding the research process and rules for open dialogue), possibility to extract data, and possibility to delete platform content after closing the project. Although less widely used in Belgium, Discord (https://www.discord.com) offered integration of voice-based and text-based options, as well as the greatest level of anonymity; users could join with a “secondary” identity and sign up with simply an email address that would stay hidden from others. To make our final decision, we asked some focus group participants about Discord vs. Slack. We ultimately selected Discord. To address lack of familiarity and give people a sense of what they might be signing up for, our invitation letter described why we chose Discord and linked to a short orientation video (that we created in both Dutch and French) with basic guidance on how to orient oneself in the platform and where to engage in dialogue.

Our Discord server *Platform for Vaccine Dialogue* was launched with a general channel for questions about Discord. A few other initial channels were for discussion topics and rules of conduct; these channels were available in Dutch and in French. Both languages are the main official languages spoken in the regions in which the HCWs needed to work. All project activities were offered in both languages to be inclusive, by giving participants the flexibility to communicate in their language(s) of choice. A feature of Discord that was also valued by the team was that Dutch and French were available as languages in the user's settings.

The platform was active from January 13 to February 21, 2022. To recruit users, we invited participants from the focus group discussions and interviews that had already taken place, and we also issued an open invitation to other HCWs and healthcare institutions. Twelve HCWs anonymously joined the Discord server. To provide food for dialogue in the server, we conducted social media analysis and posted the results weekly (e.g., sharing a word cloud with the most used hashtags from Belgian Twitter users' vaccination-related tweets that week, both in Dutch and French). As is common with online groups, a smaller subset (five users) actively posted and/or reacted to others. From the later focus group discussions, we learned that some members were reading the posts but not actively engaging.

In the Discord server, we also announced opportunities for face-to-face dialogue sessions that were prescheduled group video calls, offered separately in Dutch and French. Ten HCWs joined the group video calls during that time, half in each language.

We conducted short, Google form-based, pre- and post-intervention surveys to obtain feedback on how participants experienced different activities in this project. The pre-intervention survey was sent to all participants of the focus groups and interviews that had already taken place and to all platform users when they joined; we had 53 respondents. After the platform closed, the post-intervention survey was sent to all participants we had contact with throughout the project, not just to platform users; we had 29 respondents (including nine Discord users and five group video call participants). Roughly half of those respondents (15 out of 29) indicated they had participated in at least two project activities (i.e. focus group discussions, interviews, text-based platform, group video calls). Among those who had not joined the platform, the most frequently cited reason for non-participation was lack of time.

The seven members of our research team who were directly involved with participants regularly reflected (individually and collectively) on their experiences throughout the research and intervention design process. This was documented in weekly and *ad hoc* memos, from which the following three lessons were drawn.

## 3. Lessons

### 3.1. What dialogue means, entails, and requires may vary. (Re)Determine how the population can be (re)engaged in dialogue in an evolving context.

We had hypothesized that for open dialogue, participants would need to firstly feel safe by having an anonymous identity (i.e., not having their faces, names, and voices revealed to others) and knowing that the platform they enter would be respectful toward all speakers. We envisioned a text-based digital platform with minimal monitoring (e.g., to prevent hate speech) to be the most suitable kind of space for this. We discovered, however, that we would need to expand our approach and understanding of what open dialogue means, entails, and requires.

During the pre-intervention design period, we referred to this platform as a “safe space for dialogue” when speaking to participants; one of the first surprises was that this term could have a negative connotation. This inverted our notion of “safety.” Some participants critically asked who was really meant to be protected by these safe spaces. For them, the idea of holding private, small group discussions, centered around anonymity and confidentiality, might be less about offering a safe space for them and more about protecting the broader public from their viewpoints and ideas. For example, one participant said he considered these safe spaces as “discussions in a cellar” away from others. This led us to avoid the term “safe space” when naming our platform. Although safe spaces have garnered significant attention in the academic and activist domains (26), our results highlight how divergent understandings of this concept may drive some people away from engaging in dialogue.

Some participants had safety-related concerns that were not only about having anonymity but also about having protection from perceived untrustworthy information. They were concerned about being exposed to perceived “unscientific” content or other posts on the text-based platform that they did not consider to be “evidence-based.” For vaccine-confident participants, a safe space meant being able to block out misinformation or disinformation and knowing that there would be fact-checking of all posts.

Safety-related concerns also extended beyond the immediate digital space. Most participants described a safe space as a place where individuals could share their thoughts without fearing consequences, which meant knowing who would own and have access to their input and data. This was especially important because of the perceived risk of expressing their views (e.g., potential repercussions in the workplace when exposed).

What drew participants to our research project was the opportunity for more meaningful or authentic forms of dialogue, even if that meant less anonymity. In a polarizing context, people may be inhibited from speaking openly, but also they may have not had opportunities to have the quality of dialogue that they would make time to engage in. While vaccine hesitant participants who leaned toward pro-vaccination valued a text-based platform they could consult for reliable information, the more hesitant participants placed greater value on synchronous dialogue *via* video-based interactions, in other words, a face-to-face digital platform. For those participants, face-to-face was considered safer, because it does not enable trolling as a text-based platform does (27). Furthermore, it would allow them to see each other, to see emotions, and to evaluate the quality, intensity, and perceived trustworthiness of what others were sharing. Although having an anonymous identity was highly important, several participants still preferred face-to-face dialogue as long as they could safely use pseudonyms. Some of these participants even considered an in-person group meeting to be a safer space than a digital platform, as they believed that identities and written text could leak more easily through online engagement. Due to the Delta and Omicron waves, we could not expand to in-person interactions, but we were able to invite users to engage in group video calls (with cameras being voluntary). Based on post-intervention interviews and survey results, we concluded that text-based dialogue was not as successful as we had anticipated and that “live” face-to-face dialogue had made a bigger impact on users.

### 3.2. The cultivation of open dialogue entails a tension between helping participants voice and overcome their concerns (e.g., about vaccination) and exposing participants to others' ideas that may exacerbate those concerns. This is the paradox of open dialogue that must be adaptively navigated.

Our aim was to cultivate open dialogue that would not only give voice to diverse viewpoints but also more specifically, give voice to healthcare workers as a way to help them overcome their concerns. However, with open dialogue, there was also a risk of creating an echo chamber of narratives that might prevent the intervention from possibly contributing to building vaccine confidence, another aim of our project. This occurrence is what we call the paradox of open dialogue. In pre-intervention focus group discussions, when we encountered instances where healthcare specialists monopolized the dialogue with no pushback from other participants, the team had to reflect further on the meaning of open dialogue and the limits of free speech.

Monitoring and moderating dialogue is one way to navigate this paradox, but it is not straightforward. Because some users (particularly vaccine skeptical ones) might perceive the space as being an extension of institutional sources of information (e.g., World Health Organization) or as being another platform for debunking alternative views, we had to recognize that too strict management of the dialogue might discourage them from openly voicing their views. For vaccine hesitant HCWs, having a rule where people had to listen and respect each other's opinions brought relief. Being allowed to post articles about concerns that are usually interpreted (on other platforms) as misinformation also meant a great deal to them.

Our preparation for navigating the paradox on the text-based platform included: (1) sharing rules of conduct with users and (2) close monitoring to address imbalanced dialogue. Additionally, our risk mitigation options included, for example, recruiting more participants to help balance the dialogue and using the face-to-face dialogue sessions to address any behavioral issues observed in the text-based platform. This means that the management of a safe digital space may require drawing on different types of resources as needed, which requires ongoing attention and flexibility.

For our face-to-face dialogue sessions, navigating the paradox meant: (1) excluding the healthcare specialists who had previously hijacked the focus group discussions, which inhibited dialogue and (2) selecting a professional facilitator who generated dialogue through structured debate. The facilitator we recruited used the deep democracy approach to group dialogue and conflict management (28), which was recommended by some of our participants. The debate question was centered on the mandatory vaccination of HCWs, making use of polarization in that there were two sides. Each participant (including our team members who attended) was asked to give arguments for both sides, in order to collectively cultivate empathic dialogue.

As researchers, we hesitated to play both sides of the debate, primarily due to the potential impact that we—as researchers giving arguments against vaccination—might have on hesitant HCWs. In one session, our team members declined to give arguments against vaccination. In another session, our other team members fully participated, and this appeared to be appreciated by their participants and facilitator.

### 3.3. Interactional exchanges (e.g., with peers or experts) that matter to participants may shape the dialogue in terms of its content and form. Uncover what is relevant to participants.

In our context of vaccination among HCWs, the level of expertise or power was a key characteristic of exchanges that shaped how participants wanted to engage in dialogue and with whom (29). Notably, several participants called for what we would characterize as an “epistemically vertical” exchange, in that they were requesting information and guidance from experts. Not all participants, however, would consider a space to be safe if there was the presence of an expert (or someone who thinks they know better or who has the “official answer”). And for others, experts were seen as listeners who could make a difference; for example, some participants wanted to be heard specifically by scientific experts or others with authority, such as policymakers. Hierarchy among HCWs can also matter, for example, when doctors or specialists made strong claims and other HCWs did not push back. Reflecting a more “epistemically horizontal” exchange, some participants spoke of other HCWs as peers and preferred a facilitated dialogue among peers; they further suggested that peers be screened for their willingness to engage in dialogue.

We considered how we might incorporate all these different types of exchanges, but due to the short duration of the platform, we ultimately chose not to engage scientific experts. We believed that this could have exacerbated asymmetries in expertise and power, which would have required more time for adaptation, as the notion of an “expert” can vary based on the participant and interaction context.

Not surprisingly, a moderator or facilitator—representing a more “neutral” exchange—mattered to participants too. As researchers, we aim to preserve a certain “neutral” and trusting relationship with participants throughout the process. The polarizing nature of the topic—and thus the potential for unintentionally producing a context of “right” or “wrong” information that could shape people's vaccination decisions—pushed the team to have a clear strategy about its role. For example, if we had specifically taken on the role of an expert while conducting interviews, then the combination of asking participants for their views and sharing our “expert” view could have damaged our own epistemic position in the research process. We did not feel that we could or should serve as experts who provide “the truth” or “the right” information, because we recognized that what can be considered information vs. misinformation is not always clear.

Participants did not want to enter a space where they were to be persuaded to be pro-vaccination, but they did appreciate us posting weekly results of our social media analysis. We were careful about how we framed the posts, in order to avoid conveying our pro-vaccination stance or influencing participants in unanticipated ways. We became sensitized to this very early on, when some participants pointed out the pro-vaccination bias that they could detect in our pre-intervention survey questions (which used closed questions and a Likert scale for quantitative evaluation).

## 4. Discussion

Our case contributes a more dynamic and contextualized view to literature on addressing vaccine hesitancy with dialogue (30) and vaccine hesitancy among HCWs (31). We gained a sense of how highly contextualized and adaptive the development process for dialogue-based interventions needs to be, if we are to seriously orient to participants (e.g., not see them as simply being “users” with preferences but as also being shaped by situated meanings, paradoxes, and types of exchanges) and if we are to make use of digital platforms, which cannot equally serve all stakeholders. We do not suggest that our specific adaptations are necessarily solutions for other interventions, but rather that our adaptations reflect the need to design dialogue-based interventions with our three context-related lessons in mind.

We also suggest that having a discovery-orientation—meaning to work not only inductively and iteratively but also reflexively (e.g., where researchers are attuned to their own challenges, open to learning about their own role in shaping context, and exploring their capacity to adapt with participants)—should be a necessary part of the development of dialogue-based interventions and possibly also a part of the ongoing intervention (32, 33). Such reflexivity is lacking not only in dialogue-based interventions but also in digital health interventions (30, 34). In both types, the recursive relationships between researcher and context and between researcher and participants tend to go unacknowledged, except through mentions of researchers' limitations. What seems to run counter to a discovery-orientation is the growing interest in an approach that sits at the intersection of both dialogue-based and digital interventions: chatbots (13–15, 35, 36). Even though chatbots are considered promising, easily scalable, and adaptable, they are limited in how they can respond to rapidly-changing vaccination concerns and emotional statements (15) and thus, how they can incorporate context and cultivate dialogue. Furthermore, chatbots might not serve populations for whom authenticity of dialogue and safety of data are key requirements for engagement.

Our specific case demonstrates the importance of maintaining a discovery-orientation not only through offering three key lessons but also through shedding light on influences between: *dialogue topic*/*content* (i.e., COVID-19 vaccination), *socio-political landscape* (i.e., COVID-19 “infodemic,” fifth wave of infections nation-wide, and mandatory vaccination for HCWs), *population* (i.e., HCWs in Belgium with varying degrees of vaccine hesitancy/confidence), *intervention aim* (i.e., “safe space” for dialogue), *dialogue form* (e.g., digital, text-based, face-to-face), *ethics* (e.g., anonymity, risk of offline impact, risk of increasing vaccination concerns), *researcher position* (i.e., pro-vaccination stance, “neutral” project role, rules moderator, source of expertise or “truth”), and *types of interactional exchanges* (e.g., with healthcare peers/experts, co-workers, institutions, scientific experts, facilitators, research team). These linkages are avenues for future research. Juggling these considerations and feeling more constrained about what we could say to participants about our own views or concerns, we were also caught up in a form of “unspoken hesitancy” (24). We could not simply cultivate dialogue “from the outside” but were intertwined in the process and thus were cultivating it “from the inside.”

To advance practice, we offer three specific recommendations for how dialogue-based interventions can embrace context, and we elaborate on how these apply to the specific topic of vaccine hesitancy (Table 1). These recommendations reflect our conclusion that researchers, public health stakeholders, and other organizers should continue developing dialogue-based interventions and digital interventions in inductive and participatory ways, but with greater attention to how their own roles in an evolving context are shaping dialogue, participants, and the intervention process itself.

**Table 1:** Recommendations for more context-sensitive dialogue-based intervention design.

**Recommendations for intervention organizers**	**Application to the topic of vaccine hesitancy**
In exploring user needs during the pre-intervention design stage, be open to understanding the multiple ways in which participants may understand key concepts such as “safe dialogue” (and any related concerns)	In cases where participants need to feel safer to engage or want more meaningful dialogue, explore how their understandings of these concepts translate into viable formats for dialogue. If possible, work with participants to explore how to handle dialogue “risk factors” that shape context, such as the participation of those seen as having polarizing views or “expert” views. Other factors may include pressures to comply to directives in the workplace or hesitancy to share their concerns
Consider whether and how multiple dialogue-based interventions could be implemented, in order to reach a larger population and be more adaptive to different (and dynamic) needs	Because the framing of an intervention and its goals may not work for all participants (e.g., those who might not trust public health institutions), multiple interventions with distinct goals may need to be implemented to allow for different contexts for dialogue. For example, one intervention may explicitly aim to address information concerns while another cultivates empathy and allows for meaningful discussion
Formally integrate reflexive practices into the intervention design process, through making the time and space to discuss and respond to the emerging relationship between organizers and participants, on an ongoing basis or at key points in time	As concerns about vaccine hesitancy have been well-documented in literature, use this knowledge as a starting point to reflect on the influences and roles of the organizers. If possible, provide participants with opportunities to share not just their experience engaging in dialogue but also their experience relating to the organizers, in order to adapt to their context in a timely manner

## Data availability statement

The datasets presented in this article are not readily available because they are composed of full transcripts of several thousand words each, which even when anonymized could lead to the identification of participants. Requests to access the datasets should be directed at: the Institutional Review Board at the Institute for Tropical Medicine (Antwerp, Belgium) at irb@itg.be.

## Ethics statement

The studies involving human participants were reviewed and approved by the Institutional Review Board of the Institute of Tropical Medicine (ITM) Antwerp and the Social and Societal Ethics Committee (SMEC) of KU Leuven. The patients/participants provided their written informed consent to participate in this study.

## Author contributions

LH, CG, CVR, HL, TG-V, KPG, and NV conceptualized the project. LH, SD, HD, TN, CVR, LB, and CG conducted the research and investigation process. NV, TG-V, KPG, and HL provided oversight and leadership. TN first drafted the manuscript. LB reviewed the literature used in this paper. TN, LB, LH, CVR, CG, SD, NV, TG-V, KPG, and HL contributed to reviewing and editing of the manuscript. All authors contributed to the article and approved the submitted version.
